# Post-Stroke Depression: Impact of Lesion Location and Methodological Limitations—A Topical Review

**DOI:** 10.3389/fneur.2017.00498

**Published:** 2017-09-21

**Authors:** Alina Nickel, Götz Thomalla

**Affiliations:** ^1^Department of Neurology, Head and Neurocenter, University Medical Center Hamburg-Eppendorf, Hamburg, Germany

**Keywords:** ischemic stroke, post-stroke depression, lesion location, lesion symptom mapping, vascular depression, rehabilitation

## Abstract

Post-stroke depression (PSD) affects approximately one-third of all stroke patients. It hinders rehabilitation and is associated with worse functional outcome and increased mortality. Since the identification of PSD is a significant clinical problem, clinicians and researchers have tried to identify predictors that indicate patients at risk of developing PSD. This also includes the research question whether there is an association between PSD and stroke lesion characteristics, e.g., lesion size and lesion location. Early studies addressing this question are largely limited by technical constraints and, thus, focused on simple lesion characteristics such as lesion side or proximity of the lesion to the frontal pole of the brain. More recent studies have addressed the impact of involvement of specific neuronal circuits in the stroke lesion. State-of-the-art methods of lesion symptom mapping to study PSD have only been applied to small patient samples. Overall, results are controversial and no clear pattern of stroke lesions associated with PSD has emerged, though there are findings suggesting that more frontal stroke lesions are associated with higher incidence of PSD. Available studies are hampered by methodological limitations, including drawbacks of lesion analysis methods, small sample size, and the issue of patient selection. These limitations together with differences in approaches to assess PSD and in methods of image analysis limit the comparability of results from different studies. To summarize, as of today no definite association between lesion location and PSD can be ascertained and the understanding of PSD rests incomplete. Further insights are expected from the use of modern lesion inference analysis methods in larger patient samples taking into account standardized assessment of possible confounding parameters, such as stroke treatment and reperfusion status.

## Introduction

Post-stroke depression (PSD) is a frequently observed condition in the weeks and months following an acute stroke. Reported rates of PSD range between 11% (1) and 52% (2). Most commonly, PSD is defined as a depression that was not existent before the stroke and occurred in chronological context to a stroke. PSD is known to affect almost one in three stroke patients (3, 4). It is usually observed in a rather early time period following stroke but also as a disease that can last up to 10 years after stroke with a high risk of chronic depression (4). In a sample of patients with PSD, 1 year after stroke 40% still suffered from symptoms of depression (5).

Post-stroke depression interferes with rehabilitation from stroke, negatively affects quality of life, and may be responsible for poor outcome of stroke. Unfavorable outcomes induced by PSD involve persisting disability after stroke, mood disorders, and even increased mortality (6). A correlation between depression and mortality was observed, both for stroke patients younger than 65 years (7) and for older patients (8).

The question of predictors of PSD is of great scientific and clinical interest. Early identification of patients at risk of developing PSD might enable targeted preventive actions or early initiation of effective antidepressant treatment. This has led to scientific interest in the possible association of stroke lesion characteristics, mostly lesion location, and the occurrence of PSD. In this review, we provide an overview of the current research on this topic. We not only focus on the association of stroke lesion characteristics and PSD but also touch clinical parameters and methodological aspects.

## Methods

This is a narrative review. As a basis for a selective literature review, we performed an extensive literature research. We searched PubMed using the search terms “lesion location AND post-stroke depression” OR “lesion location AND depression AND stroke.” This resulted in 159 publications of which 12 were selected according to criteria outlined below. We also searched references from a recent systematic review on PSD (9) and screened further reviews on PSD for additional publications addressing the impact of lesion location. In order to identify more recent publications not yet included in the review by Wei et al. (9), we performed another PubMed search using the search terms “depression AND stroke AND mapping AND lesion ” and the filter “from 01.01.2014 until today [12.06.2017]” which resulted in five further publications, of which we included four in our review.

We included studies in our review that met the following criteria: (1) published in English, (2) reporting results on the association between lesion location and PSD, (3) providing information on the criteria for diagnosing depression or the rating scale employed and the methods to determine lesion location, (4) determination of stroke lesions on CT or MRI, and (5) providing data of a minimum of 50 stroke patients. We also included selected publications that did not meet these criteria if they contain a relevant new methodological approach or report findings of special interest. Publications reporting duplicate results or case reports were excluded.

## Clinical Predictors of PSD

A number of clinical parameters were reported to be associated with PSD: higher age (10, 11) but also younger age (25), stroke severity, and physical disability resulting from stroke (2, 4–6, 10, 12, 14–16) (see Table 1 for a summary of most important clinical predictors of PSD). Cognitive impairment resulting from stroke (2, 4, 6, 10, 14, 15) as well as specific disabling stroke symptoms such as aphasia (5, 14) or apraxia (15) also correlated with PSD. Further predictors of PSD comprise a history of psychiatric disease, especially previous depression (4, 6, 12, 18), but also social isolation (5, 6, 10, 14), level of education (13, 17, 19), institutionalization after stroke (10), or stressful life events before stroke (12, 13).

**Table 1:** Predictors of post-stroke depression.

Topics	Predictors	Frequency and reported strength of predictor
Demographics	Higher age	Only mentioned in few studies, weak predictor (10, 11)
	Female sex	Mentioned in several studies (6, 10, 12, 13)

Stroke characteristics	Severity	Mentioned in several studies (4, 6, 11, 14)

Stroke outcome	Physical disability	Mentioned in many studies, strong predictor (2, 4–6, 10, 12, 14–16)
	Intellectual impairment	Mentioned in many studies, strong predictor (2, 4, 6, 10, 14, 15)
	Dysphasia	Only mentioned in few studies, weak predictor (5, 14)
	Apraxia	Only mentioned in few studies, weak predictor (14)

Vascular risk factors	E.g., hypertension	Mentioned in several studies (6, 15, 17)

History/pre-existing diseases	Psychiatric disease before pre-stroke	Mentioned in many studies, strong predictor (4, 6, 12, 18)
	Former stroke	Only mentioned in few studies, weak predictor (12)
	Cerebral atrophy	Only mentioned in few studies, weak predictor (5)
	Pathological crying	Only mentioned in few studies, weak predictor (12)

Life events/social situation	Isolation/quality of social support	Mentioned in several studies (5, 6, 10, 14)
	Residence in an institution	Only mentioned in few studies, weak predictor (10)
	Level of education	Mentioned in several studies (13, 17, 19)
	Stressful life event pre-stroke	Only mentioned in few studies, weak predictor (12, 13)

## How to Diagnose PSD?

Gold standard for the diagnosis of depression represents the DSM-IV and DSM-V criteria. Available studies used quite heterogeneous approaches to screen for PSD as well as to quantify depressive symptoms of PSD. The following established diagnostic tests were used most frequently for the detection of PSD in the studies recited in this review: the Hamilton rating scale for depression (HAMD) contains 21 items that are judged by an examiner and is regarded as an indication of depression (26). The Beck Depression Inventory (BDI) comprises 21 self-rating questions and assesses depression severity (27). The Zung self-rating depression scale consists of 20 items (28). Finally, the Montgomery–Åsberg Depression Rating Scale (MADRS) consists of 10 questions and is filled out by an examiner (29). Each of these scales provides a cutoff that defines depression but also enables continuous evaluation of symptoms assessed by the scale, and both approaches have been used in studies of PSD. BDI and HAMD showed adequate testing results in stroke patients compared to the DSM-IV (30), as well as the MADRS (31). The Zung self-rating depression scale was compared to other screening methods in geriatric stroke patients and assessed with a high positive predictive value (32). The Patient Health Questionnaire-9 (PHQ-9) is another frequently used scale for screening for depressive symptoms proven to be reliable in stroke patients (33).

## PSD and the Vascular Depression Hypothesis

The hypothesis of “vascular depression” refers to the concept of vulnerability to depressive symptoms resulting from structural brain damage caused by cerebrovascular disease mainly in elderly people (34). The proposal of vascular depression as a distinct subtype of depression resulted from clinical observations of late-onset depression being accompanied by co-morbid impairment of executive functions and findings of white matter hyperintensities (WMH) on T2-weighted MRI as compared to early-onset depressive disorder.

In addition to brain WMH assumed to reflect small vessel disease, multiple infarcts of the brain were described as cause of VD (35). There are two hypotheses as to the relation between brain lesions and depression in vascular depression. One is that VD is due to the overall load of small vessel disease with many silent brain infarctions and that with a certain “threshold” amount, VD is triggered. The other hypothesis is that vascular damage from lesions in specific strategic locations leads to VD (36). As the second hypothesis resembles the lesion location hypothesis of PSD, the connection of these two phenomena will be discussed here. Some researchers even argue that PSD is a kind of VD because both diseases involve depressive symptoms that are both symptom and cause of CVD (37).

However, when comparing PSD and VD, similarities as well as differences arise. On the one hand, both syndromes are due to ischemic brain damage, both can lead to cognitive impairment, increase mortality, and occur more frequently in elderly patients. On the other hand, VD rather affects small vessels and “silent brain infarctions” can be seen; whereas in PSD, the patient, by definition of stroke, is symptomatic and often larger brain vessels are affected. The risk of PSD events seems to correlate with stroke severity. Furthermore, when looking at the type of depressive disorder, PSD appears more to act like an endogenous depression than VD (38).

Two studies that were included in this review, investigated not only the relationship between lesion location and PSD but also the existence of WMH in stroke patients (39, 40). They evaluated deep WMH (DWMH) and periventricular WMH (PWMH) and observed no significant relationship between WMH and the presence of depressive symptoms. By contrast, another recent study reported a significant correlation between markers of small vessel disease, i.e., WMH and asymptomatic lacunar infarcts, and PSD (41). Brain network damage affecting fronto-limbic circuits might represent the common mechanism between small vessel disease and white matter damage linked to VD, on the one hand, and PSD after singular stroke lesions, but as of today this has not been further studied.

## Different Symptoms and Clinical Subtypes of PSD

There are several symptoms of depression that can become manifest after stroke, and different studies have put the focus on different symptoms of PSD, which may partly explain the resulting varying conclusions as to the relation of stroke lesions and PSD. Anhedonia, defined as the inability to enjoy normally enjoyable activities was reported to be correlated with stroke lesion volume in the parahippocampal gyrus, and the authors suggested that stroke lesions in this region lead to disruption in the hypothalamic–pituitary–adrenal axis control cycle (42).

Apathy as symptom of depression defined as “an absence or a decrease in motivations, interests, and emotions, which cannot be ascribed to a lack of consciousness, cognitive impairment, or emotional distress” was also addressed (43). An association between apathy and right hemispheric stroke in the basal ganglia was observed (44). According to a review on the issue apathy is more frequent after stroke than depression and that apathetic patients are more affected in terms of cognitive impairment and depressive symptoms (45). The differentiation between affective-depressive and apathetic subtypes of PSD goes into the same direction. A study using this differentiation reports that severity of affective depression was associated with left frontal lobe damage while that of apathetic depression was linked to bilateral basal ganglia damage (46). A recent study investigated the association of stroke location and apathy using diffusion tensor imaging (DTI) (47), which is described below.

## Association of Stroke Lesion with PSD

The idea of an association of stroke lesions in specific brain areas to PSD traces back to the beginning of the twentieth century and the observation of focal damage to the brain leading to psychiatric symptoms (48). In 1977, a link of mood disorders after stroke to right hemispheric stroke was suggested for the first time based on the observation of 20 stroke patients (49). In the years to follow, the search for a stroke location linked to depression became more precise, following the development of methods of stroke imaging and lesion analysis. Initially, a rather rough anatomic allocation of stroke lesions was used to analyze lesion behavior mapping with PSD, i.e., left versus right hemispheric stroke, involvement of the frontal lobe, or location of stroke lesions following an anterior–posterior gradient. Recently, increased understanding of brain networks involved in the pathophysiology of depression (50) motivated more refined anatomic interpretation of stroke lesion location linked to PSD. Furthermore, severity of PSD was reported to be associated with lesion location (51, 52) but not with lesion size (52).

Most available studies of PSD have used rather basic methods in order to assess the association of stroke lesion with PSD. Only recently, modern approaches of localizing stroke lesions with reference to standard brain atlases and of voxel-based analysis have been applied in studies of PSD. An overview of reviews that examined the correlation of PSD and lesion location and their results is given by Table 2.

**Table 2:** Reviews on post-stroke depression (PSD) and lesion location and main results.

Year	Reference	Numbers of studies included in review/meta-analysis	Results—association of stroke lesions with PSD?
2000	Carson et al. (20)	48	No
2003	Narushima et al. (21)	27	Yes, proximity frontal pole
2004	Yu et al. (22)	52	(Yes) right
2006	Spalletta et al. (23)	109	Yes, limbic areas
2014	Kutlubaev et al. (24)	23	No
2015	Wei et al. (9)	43	Yes, right
2016	Robinson and Jorge (6)	No information on number of included studies	Yes, left frontal/basal ganglia

Lesion volume is a predictor of stroke outcome in general, and given the association of stroke severity with PSD, a possible association of stroke lesion volume with PSD is well imaginable. There are, however, contradictory findings. While some studies report a correlation between lesion volume and severity of depression (1, 2, 10, 39, 40, 53, 54), no correlation between lesion volume and PSD was observed in other studies (5, 55). To the effect of the interaction between lesion location and lesion volume on PSD, contradictory findings were reported. For instance, an impact of lesion volume on PSD was stated for lesion located in the left anterior hemisphere (53), the right hemisphere (54), and in the left limbic–cortical–striatal–pallidal–thalamic circuit (39).

## Lesion Location—Visual Analysis

### Laterality

Laterality was one of the first characteristics of stroke lesions reported to be linked to symptoms of PSD with symptoms of depression observed more frequently in right hemispheric (22, 49, 54, 56). There are, however, studies with contradictory finding, i.e., an association of left hemispheric lesions with higher prevalence and severity of PSD (5, 11, 57).

### Lesion Location across an Anterior–Posterior Gradient

Proximity of the stroke lesion to the frontal pole was examined as an early approach to relate PSD to stroke lesion location, and more frontal location of stroke lesions was associated with higher rates of PSD (53, 58), a finding just recently reproduced in a large sample of stroke patients (59).

### Involvement of Frontal–Subcortical Circuits

A first study of PSD describing lesion location more detailed by discriminating between cortical, subcortical, brain stem, cerebellar, and hemorrhagic stroke was published in 1983 (60). Further studies supported a correlation between cortical and subcortical stroke lesions in the left anterior lobe and increased frequency and severity of depression (13, 61). Later studies addressed the involvement of specific brain areas as parts of neuronal circuits in the context of PSD and observed a correlation between PSD and lesions in frontal–subcortical circuits, including pallidum and nucleus caudate (62). In a similar manner, stroke lesions in the amygdala, medial prefrontal cortex, striatum, pallidum, and thalamus associated with PSD were interpreted as lesions affecting frontal cortico-limbic circuits (17, 39, 40).

Almost all of the listed studies used visual analysis of CT or MRI images. Only one group of researchers performed the analyses more systematically *via* normalization and registration of the stroke lesions to the Brodmann’s Cytoarchitectonic Atlas and Statistical Parametric Mapping (39). An overview of studies using a classical visual approach to determine lesion location and the association with PSD is given in Table 3.

**Table 3:** Studies using visual judgment for the analysis of lesion location and PSD.

Reference	Sample size	Depressed (%)	Imaging technique/analysis method	Result (lesion localization associated with PSD)
Robinson and Jorge (57)	103	30 (29)	CT/visual	Left frontal
				Proximal frontal pole
House et al. (1)	73	8 (11)	CT/visual	No association
Aström et al. (5)	80	(25)	CT/visual	Left frontal
Morris et al. (55)	193	74 (38)	CT/visual	No association
MacHale et al. (54)	55	11 (20)	CT/visual	Right hemisphere
Berg et al. (11)	100	27 (27)	CT, MRI/visual	Left hemisphere
				Brain stem
Vataja et al. (62)	70	26 (37)	MRI/visual	Frontal–subcortical circuit
Nys et al. (2)	126	65 (52)	CT, MRI/visual	No association
Tang et al. (13)	189	31 (16)	CT/visual	Subcortical
				ACA territory
Hama et al. (46)	243	126 (52)	CT/visual	Left frontal
Snaphaan et al. (16)	420	54 (13)	CT, MRI/visual	No association
Nishiyama et al. (17)	134	45 (34)	CT, MRI/visual	Left lenticulocapsular
Castellanos-Pinedo et al. (18)	98	21 (24)	CT, MRI/visual	Right hemisphere
Zhang et al. (40)	163	39 (24)	MRI/visual	Frontal/temporal lobe
				Internal capsule
Shi et al. (59)	1067	303 (28)	CT, MRI/visual	Frontal lobe
Metoki et al. (56)	421	71 (17)	MRI/visual	Right frontal and temporal
Guiraud et al. (12)	251	61 (24)	CT, MRI/visual	No significant association

*PSD, post-stroke depression*.

In contrast to the large number of studies mentioned before, there is also a considerable number of studies questioning a relationship between PSD and stroke lesion location using different approaches to lesion analysis (1, 21, 55). This notion was supported by early reviews on the issue of lesion location and PSD which concluded that no reliable association of stroke lesion location and PSD could be established based on the available results (20, 63). Possible reasons for this may be heterogeneity and limitations in methods used to assess depression and lesion location as well as small number of patients included in most studies. A further discussion of methodological limitations of the available studies can be found below.

## Voxel-Based Lesion Symptom Mapping (VLSM) of PSD

Only in 2014, to our knowledge, the first study applied voxel-based symptom lesion mapping (VLSM) in PSD research (64), while the method was introduced into stroke research already at the beginning of the twenty-first century. Developed by Rorden and Brett (65), VLSM involves lesion segmentation on high-resolution brain imaging, includes registration of individual brain images to standard space and, finally, statistical testing to identify voxels that if affected by the lesion are significantly associated with clinical characteristics. There are, however, some statistical constraints that need to be considered when VLSM is performed. An adequate sample size with a sufficient overlap between lesions is required to enable reliable statistics, as the usually applied Brunner–Munzel test requires voxels to be affected in at least 10 subjects to be carried out appropriately (66).

In a pilot VLSM study of PSD, 27% of 55 patients met criteria for depression 1 month after stroke, but no voxels or clusters were significantly associated with PSD (64). The authors acknowledge methodological limitations that might explain the lack of an association between lesion localization and depression in their study, including only minimal overlap in lesion distribution between the groups and the early time point of clinical assessment. A second recently published VSLM study specifically investigated the relationship between isolated cerebellar stroke and PSD in a small sample of 24 patients, of whom eight were diagnosed with PSD within the first 3 months of stroke (52). Stroke lesions in the posterior lobe of the left cerebellar hemisphere and the left cerebellar peduncle were significantly correlated with severity of depression. According to the interpretation of the authors, these findings add to the understanding of the role of the cerebellum in emotional processing.

Another inherent limitation of VLSM is the mass univariate approach that makes the method vulnerable to consistent errors, i.e., consistent lesion patterns of brain areas that lesioned together resulting from anatomy of vascular supply to the brain while being functionally segregated (67). With regard to this limitation, a third VLSM study of PSD is interesting, as they applied multivariate analysis using on support vector regression-based lesion symptom mapping (LSM), an approach that may overcome the limitations of univariate analysis. In this study, the authors investigated the relationship between lesion location and PSD in both aphasic and non-aphasic stroke patients (*n* = 39) using the Stroke Aphasic Depression Questionnaire (SADQ) (19). Stroke lesions in the left dorsolateral prefrontal cortex were significantly associated with the severity of depressive symptoms. Table 4 provides a summary of studies using modern methods of lesion inference analysis.

**Table 4:** Studies using modern methods of lesion inference analysis.

Reference	Sample size	Depressed (%)	Imaging technique/analysis method	Result (lesion localization associated with post-stroke depression)
Terroni et al. (39)	68	21 (31)	MRI/statistical mapping	Left cortical
				Limbic–cortical–striatal–pallidal–thalamic circuit
Gozzi et al. (64)	55	15 (27)	MRI/VLSM	No association
Hama et al. (68)	48	14 (29)	MRI/statistical mapping	Basal ganglia
Kim et al. (52)	24	8 (33)	MRI/VLSM	Left posterior cerebellar hemisphere
Grajny et al. (19)	39	Continuous score	MRI/multivariate lesion symptom mapping	Left DLPFC

*VLSM, voxel-based lesion symptom mapping; DLPFC, dorsolateral prefrontal cortex*.

Thus, as of today, only three small studies with sample size ranging between 24 and 55 used VLSM to study the association of depressive symptoms following stroke with lesion location. Moreover, one of these studies selectively looked at isolated cerebellar strokes. Altogether, these studies were largely underpowered and are far from being conclusive. They can mainly be considered as pilot studies that may stimulate further research.

Lesion symptom mapping may also be applied to examine the relation of stroke lesions to metabolites of neurotransmitters in order to examine possible pathophysiological mechanisms of PSD. In a pilot study following this approach, the authors performed statistical parametric mapping of several neurotransmitters and their metabolites quantified in blood or urine samples and identified specific patterns for the different neurotransmitters (68). While brainstem lesions were related to reduced noradrenaline and dopamine levels in urine samples, cortical and striatal lesions were associated with increased levels of noradrenaline and dopamine. By contrast, serotonine and its metabolites showed a more or less opposite pattern, so the authors suggest that these data reflect the anatomical and functional subdivision of the monoamine network into two specific components, i.e., catecholamine (noradrenaline and dopamine) and serotonine.

## Brain Networks and PSD: A Role for DTI?

The potential role of stroke lesions affecting brain regions within specific fronto-striatal brain networks in PSD was raised by earlier studies based on visual characterization of stroke lesions as described above. Looking at the possible role of specific circuits and networks in PSD brings another technique of modern brain imaging into play that has emerged as powerful technique for the *in vivo* study of brain networks, i.e., DTI. Based on quantitative study of water diffusion properties of brain tissue, DTI enables the analysis of organizational properties of white matter fiber tracts (69). One of the most frequently used indices to describe tissue diffusion properties is fractional anisotropy (FA), which may act as an indicator of the degree of organization as well as of the integrity of brain white matter.

There is an increasing number of DTI studies in patients with depressive disorder that report findings of toward structural alterations of fronto-striato-thalamic pathways and anterior interhemispheric connections, i.e., pathways interconnecting brain regions and networks involved in tasks such as decision-making, emotional regulation, and reward-processing (70). These findings point toward a possible specific role structural networks connecting frontal and subcortical brain regions in depression that might provide a link between structural brain lesions and PSD. However, while DTI has been extensively used in psychiatric research to study structural brain changes associated with depression, we found only a single study that used DTI in the study of symptoms of depression following stroke. In this study of apathy post-stroke, 54 patients were studied, and decreased FA reflecting damage to the genu and splenium of the corpus callosum, left anterior corona radiata, and to white matter of the frontal gyrus was associated with apathy (47).

## Methodological Limitations of Available Studies on PSD

As already touched upon in the previous paragraphs, virtually all available studies on the association of stroke lesions and PSD are flawed from a number of methodological limitations. Most studies even up to very recent years rely on raw and rather outdated methods of lesion analysis, i.e., visual inspection and allocation to anatomical areas by expert judgment. Available modern techniques of lesion analysis, including lesion segmentation, methods of brain registration to standard space, voxel-based statistics such as VLSM, are only used in a very small number of recent pilot studies.

Small sample size represents another crucial limitation of the available studies making any conclusion on lesion symptom associations susceptible to chance if not rendering reliable statistical analysis completely impossible, especially against the background of heterogeneous stroke lesions with only limited overlap between lesions among patients in smaller samples.

Furthermore, exclusion of large groups of patients, e.g., aphasic patients, from numerous studies may lead to severe selection bias. Various methods of measuring depression represent another issue. There is no single established score or scale that has emerged to measure PSD, but rather a number of different instruments and scales with different focus (71). The same holds true for the time point of evaluation which varies largely between studies, ranging from the early subacute stage several weeks after stroke to the late chronic stage years after stroke. Moreover, most of the referenced studies did not clearly rule out or provide information on pre-stroke depression in patients included. Different approaches of analysis further hinder comparability of study results, e.g., group comparisons between patients with and without PSD based on cutoffs in depression scores, on the one hand, versus parametric analysis of values on depression scores, on the other hand.

## Conclusion

To summarize, no clear pattern emerges as to the association of stroke lesions and PSD, which largely results from methodological limitations of these studies. If any conclusions can be drawn at all, it appears that lesions in more frontal brain areas and lesions involving the basal ganglia are more prone to lead to PSD. Maybe patients with left hemispheric stroke are also more susceptible for PSD. Given the large number of studies, no firm conclusion can be drawn, which largely results from the methodological limitations of available studies. Examples of lesion behavior analysis in other research areas, e.g., neglect or aphasia, suggest that a deeper understanding of the potential impact of focal stroke lesions on the occurrence of depressive symptoms post-stroke may be expected from studies using modern methods of lesion inference analysis in larger samples. As of today only very few underpowered pilot studies using these techniques to study PSD have been published. Further research should also take into account analysis of damage to specific brain networks by stroke lesions. Research combining comprehensive clinical characterization, laboratory studies of neurotransmitter metabolites and brain lesions may shed light on possible pathomechanisms of PSD that might help understand why lesions to specific brain areas are likely to cause PSD whereas others are not. Given the available scientific evidence, it cannot be answered whether VD and PSD represent a spectrum of depression resulting from vascular brain damage or should be understood as different entities. Investigation of white matter damage together with focal stroke lesions may foster the understanding of possible common mechanisms of vascular depression and PSD. Future research strategies should include leukoaraiosis as important covariate in the evaluation of PSD and study the frequency and severity of PSD in patients with and without relevant pre-existing white matter lesions. The analysis of imaging and clinical data from available large databases and trial populations based on pre-specified analysis plans will likely provide further insights into the association of stroke lesions and PSD.

## Author Contributions

The authors (GT and AN) contributed substantially to the conception and design of this work and drafting of the work and revised it critically for important intellectual content. They approved of the version to be published and agreed to be accountable for all aspects of the work in ensuring that questions related to the accuracy or integrity of any part of the work are appropriately investigated and resolved.

## Conflict of Interest Statement

GT has received fees as consultant or lecturer from Acandis, Bayer, Bristol-MyersSquibb/Pfizer, Boehringer Ingelheim, Covidien, Daiichi-Sankyo, and GlaxoSmithKline. He receives funding from the European Union Seventh Framework Programme [FP7/2007-2013] under grant agreement no: 278276 (WAKE-UP) and no. 634809 (PRECIOUS) and from the German Research Foundation (DFG), SFB 936 “Multi-site Communication in the Brain” (Project C2). AN reports no conflicts of interest. The reviewer WK and handling editor declared their shared affiliation.

## References

[1] House A Dennis M Warlow C Hawton K Molyneux A. Mood disorders after stroke and their relation to lesion location. A CT scan study. Brain (1990) 113(Pt 4):1113–29. 10.1093/brain/113.4.1113 2397385

[2] Nys GMS Van Zandvoort MJE Van Der Worp HB De Haan EHF De Kort PLM Kappelle LJ. Early depressive symptoms after stroke: neuropsychological correlates and lesion characteristics. J Neurol Sci (2005) 228:27–33. 10.1016/j.jns.2004.09.031 15607207

[3] Hackett ML Yapa C Parag V Anderson CS. Frequency of depression after stroke: a systematic review of observational studies. Stroke (2005) 36:1330–40. 10.1161/01.STR.0000165928.19135.35 15879342

[4] Ayerbe L Ayis S Wolfe C Rudd A. Natural history, predictors and outcomes of depression after stroke: systematic review and meta-analysis. Br J Psychiatry (2013) 202:14–21. 10.1192/bjp.bp.111.107664 23284148

[5] Aström M Adolfsson R Asplund K. Major depression in stroke patients. A 3-year longitudinal study. Stroke (1993) 24:976–82. 10.1161/01.STR.24.7.976 8322398

[6] Robinson RG Jorge RE. Post-stroke depression: a review. Am J Psychiatry (2016) 173:221–31. 10.1176/appi.ajp.2015.15030363 26684921

[7] Ayerbe L Ayis S Crichton SL Rudd AG Wolfe CDA Explanatory factors for the increased mortality of stroke patients with depression. Neurology (2014) 83:2007–12. 10.1212/WNL.0000000000001029 25355829 PMC4248453

[8] House A Knapp P Bamford J Vail A. Mortality at 12 and 24 months after stroke may be associated with depressive symptoms at 1 month. Stroke (2001) 32:696–701. 10.1161/01.str.32.3.696 11239189

[9] Wei N Yong W Li X Zhou Y Deng M Zhu H Post-stroke depression and lesion location: a systematic review. J Neurol (2015) 262:81–90. 10.1007/s00415-014-7534-1 25308633

[10] Sharpe M Hawton K Seagroatt V Bamford J House A Molyneux A Depressive disorders in long-term survivors of stroke. Associations with demographic and social factors, functional status, and brain lesion volume. Br J Psychiatry (1994) 164:380–6. 10.1192/bjp.164.3.380 8199792

[11] Berg A Palomäki H Lehtihalmes M Lönnqvist J Kaste M. Poststroke depression in acute phase after stroke. Cerebrovasc Dis (2001) 12:14–20. 10.1159/000047675 11435674

[12] Guiraud V Gallarda T Calvet D Turc G Oppenheim C Rouillon F Depression predictors within six months of ischemic stroke: the DEPRESS study. Int J Stroke (2016) 11:1747493016632257. 10.1177/1747493016632257 26873940

[13] Tang WK Chan SSM Chiu HFK Ungvari GS Wong KS Kwok TCY Poststroke depression in Chinese patients: frequency, psychosocial, clinical, and radiological determinants. J Geriatr Psychiatry Neurol (2005) 18:45–51. 10.1177/0891988704271764 15681628

[14] De Ryck A Fransen E Brouns R Geurden M Peij D Mariën P Poststroke depression and its multifactorial nature: results from a prospective longitudinal study. J Neurol Sci (2014) 347:159–66. 10.1016/j.jns.2014.09.038 25451004

[15] Allan LM Rowan EN Thomas AJ Polvikoski TM O’Brien JT Kalaria RN. Long-term incidence of depression and predictors of depressive symptoms in older stroke survivors. Br J Psychiatry (2013) 203:453–60. 10.1192/bjp.bp.113.128355 24158880

[16] Snaphaan L Van Der Werf S Kanselaar K De Leeuw FE. Post-stroke depressive symptoms are associated with post-stroke characteristics. Cerebrovasc Dis (2009) 28:551–7. 10.1159/000247598 19844094

[17] Nishiyama Y Komaba Y Ueda M Nagayama H Amemiya S Katayama Y. Early depressive symptoms after ischemic stroke are associated with a left lenticulocapsular area lesion. J Stroke Cerebrovasc Dis (2010) 19:184–9. 10.1016/j.jstrokecerebrovasdis.2009.04.002 20434044

[18] Castellanos-Pinedo F Hernandez-Perez JM Zurdo M Rodriguez-Funez B Hernandez-Bayo JM Garcia-Fernandez C Influence of premorbid psychopathology and lesion location on affective and behavioral disorders after ischemic stroke. J Neuropsychiatr (2011) 23:340–7. 10.1176/appi.neuropsych.23.3.340 21948896

[19] Grajny K Pyata H Spiegel K Lacey EH Xing S Brophy C Depression symptoms in chronic left hemisphere stroke are related to dorsolateral prefrontal cortex damage. J Neuropsychiatry Clin Neurosci (2016) 28:292–8. 10.1176/appi.neuropsych.16010004 27255855

[20] Carson AJ MacHale S Allen K Lawrie SM Dennis M House A Depression after stroke and lesion location: a systematic review. Lancet (2000) 356:122–6. 10.1016/S0140-6736(00)02448-X 10963248

[21] Narushima K Kosier JT Robinson RG. A reappraisal of poststroke depression, intra- and inter-hemispheric lesion location using meta-analysis. J Neuropsychiatry Clin Neurosci (2003) 15:422–30. 10.1176/appi.neuropsych.15.4.422 14627768

[22] Yu L Liu C-K Chen J-W Wang S-Y Wu Y-H Yu S-H. Relationship between post-stroke depression and lesion location: a meta-analysis. Kaohsiung J Med Sci (2004) 20:372–80. 10.1016/S1607-551X(09)70173-1 15473648 PMC11918149

[23] Spalletta G Bossù P Ciaramella A Bria P Caltagirone C Robinson RG. The etiology of poststroke depression: a review of the literature and a new hypothesis involving inflammatory cytokines. Mol Psychiatry (2006) 11:984–91. 10.1038/sj.mp.4001879 16894392

[24] Kutlubaev MA Hackett ML. Part II: predictors of depression after stroke and impact of depression on stroke outcome: an updated systematic review of observational studies. Int J Stroke (2014) 9:1026–36. 10.1111/ijs.12356 25156411

[25] Carota A Berney A Aybek S Laria G Staub F Ghika-Schmid F A prospective study of predictors of poststroke depression. Neurology (2005) 64:428–33. 10.1212/01.WNL.0000150935.05940.2D 15699370

[26] Hamilton M A rating scale for depression. J Neurol Neurosurg Psychiatry (1960) 23:56–62. 10.1136/jnnp.23.1.56 14399272 PMC495331

[27] Beck AT Ward CH Mendelson M Mock J Erbaugh J An inventory for measuring depression. Arch Gen Psychiatry (1961) 4:561–71. 10.1001/archpsyc.1961.01710120031004 13688369

[28] Zung WW A self-rating depression scale. Arch Gen Psychiatry (1965) 12:63–70. 10.1001/archpsyc.1965.01720310065008 14221692

[29] Montgomery SA Asberg M. A new depression scale designed to be sensitive to change. Br J Psychiatry (1979) 134:382–9. 10.1192/bjp.134.4.382 444788

[30] Aben I Verhey F Lousberg R Lodder J Honig A. Validity of the beck depression inventory, hospital anxiety and depression scale, SCL-90, and Hamilton depression rating scale as screening instruments for depression in stroke patients. Psychosomatics (2002) 43:386–93. 10.1176/appi.psy.43.5.386 12297607

[31] Sagen U Vik TG Moum T Mørland T Finset A Dammen T. Screening for anxiety and depression after stroke: comparison of the hospital anxiety and depression scale and the Montgomery and Asberg depression rating scale. J Psychosom Res (2009) 67:325–32. 10.1016/j.jpsychores.2009.03.007 19773025

[32] Agrell B Dehlin O. Comparison of six depression rating scales in geriatric stroke patients. Stroke (1989) 20:1190–4. 10.1161/01.STR.20.9.1190 2772980

[33] Turner A Hambridge J White J Carter G Clover K Nelson L Depression screening in stroke: a comparison of alternative measures with the structured diagnostic interview for the diagnostic and statistical manual of mental disorders, fourth edition (major depressive episode) as criterion standard. Stroke (2012) 43:1000–5. 10.1161/STROKEAHA.111.643296 22363064

[34] Alexopoulos GS Meyers BS Young RC Campbell S Silbersweig D Charlson M “Vascular depression” hypothesis. Arch Gen Psychiatry (1997) 54:915–22. 10.1001/archpsyc.1997.01830220033006 9337771

[35] Krishnan KR Hays JC Blazer DG. MRI-defined vascular depression. Am J Psychiatry (1997) 154:497–501. 10.1176/ajp.154.4.497 9090336

[36] Kimura M Vascular depression. JMAJ (2004) 47:573–8.

[37] Dieguez S Staub F Bruggimann L Bogousslavsky J. Is poststroke depression a vascular depression? J Neurol Sci (2004) 226:53–8. 10.1016/j.jns.2004.09.012 15537520

[38] Volk S Steffens DC Post-stroke depression and vascular depression. In: Lavretsky H Sajatovic M Reynolds C, editors. Late-Life Mood Disorders. Oxford: Oxford University Press p. 254–69.

[39] Terroni L Amaro E Iosifescu DV Tinone G Sato JR Leite CC Stroke lesion in cortical neural circuits and post-stroke incidence of major depressive episode: a 4-month prospective study. World J Biol Psychiatry (2011) 12:539–48. 10.3109/15622975.2011.562242 21486107 PMC3279135

[40] Zhang T Jing X Zhao X Wang C Liu Z Zhou Y A prospective cohort study of lesion location and its relation to post-stroke depression among Chinese patients. J Affect Disord (2012) 136:e83–7. 10.1016/j.jad.2011.06.014 21763001

[41] Zhang X Tang Y Xie Y Ding C Xiao J Jiang X Total magnetic resonance imaging burden of cerebral small-vessel disease is associated with post-stroke depression in patients with acute lacunar stroke. Eur J Neurol (2017) 24:374–80. 10.1111/ene.13213 27933697

[42] Terroni L Amaro E Iosifescu DV Mattos P Yamamoto FI Tinone G The association of post-stroke anhedonia with salivary cortisol levels and stroke lesion in hippocampal/parahippocampal region. Neuropsychiatr Dis Treat (2015) 11:233–42. 10.2147/NDT.S73722 25678790 PMC4322890

[43] Spalletta G Cravello L Piras F Iorio M Sancesario G Marchi A Rapid-onset apathy may be the only clinical manifestation after dorsal striatum hemorrhagic lesion. Alzheimer Dis Assoc Disord (2013) 27:1 10.1097/WAD.0b013e318260ab97 22760169

[44] Finset A Andersson S. Coping strategies in patients with acquired brain injury: relationships between coping, apathy, depression and lesion location. Brain Inj (2000) 14:887–905. 10.1080/026990500445718 11076135

[45] Caeiro L Ferro JM Costa J. Apathy secondary to stroke: a systematic review and meta-analysis. Cerebrovasc Dis (2013) 35:23–39. 10.1159/000346076 23428994

[46] Hama S Yamashita H Shigenobu M Watanabe A Kurisu K Yamawaki S Post-stroke affective or apathetic depression and lesion location: left frontal lobe and bilateral basal ganglia. Eur Arch Psychiatry Clin Neurosci (2007) 257:149–52. 10.1007/s00406-006-0698-7 17131217

[47] Yang SR Shang XY Tao J Liu JY Hua P. Voxel-based analysis of fractional anisotropy in post-stroke apathy. PLoS One (2015) 10:e116168. 10.1371/journal.pone.0116168 25555189 PMC4282201

[48] Meyer A The anatomical facts and clinical varieties of traumatic insanity.Am J Insa (1904) 60(3):373–441. 10.1176/ajp.60.3.373 10956578

[49] Folstein MF Maiberger R McHugh PR. Mood disorder as a specific complication of stroke. J Neurol Neurosurg Psychiatry (1977) 40:1018–20. 10.1136/jnnp.40.10.1018 591971 PMC492887

[50] Chaudhury D Liu H Han MH. Neuronal correlates of depression. Cell Mol Life Sci (2015) 72:4825–48. 10.1007/s00018-015-2044-6 26542802 PMC4709015

[51] Robinson RG Book Starr L Lipsey JR Rao K Price TR A two-year longitudinal study of post-stroke mood disorders: dynamic changes in associated variables over the first six months of follow-up. Stroke (1984) 15:510–7. 10.1161/01.STR.15.3.510 6729881

[52] Kim NY Lee SC Shin J-C Park JE Kim YW Voxel-based lesion symptom mapping analysis of depressive mood in patients with isolated cerebellar stroke: a pilot study. Neuroimage Clin (2017) 13:39–45. 10.1016/j.nicl.2016.11.011 27942446 PMC5133641

[53] Robinson RG Kubos KL Starr LB Rao K Price TR Mood disorders in stroke patients. Brain (1984) 107:81–93. 10.1093/brain/107.1.81 6697163

[54] MacHale SM O’Rourke SJ Wardlaw JM Dennis MS. Depression and its relation to lesion location after stroke. J Neurol Neurosurg Psychiatry (1998) 64:371–4. 10.1136/jnnp.64.3.371 9527152 PMC2170020

[55] Morris PL Robinson RG de Carvalho ML Albert P Wells JC Samuels JF Lesion characteristics and depressed mood in the stroke data bank study. J Neuropsychiatry Clin Neurosci (1996) 8:153–9. 10.1176/jnp.8.2.153 9081550

[56] Metoki N Sugawara N Hagii J Saito S Shiroto H Tomita T Relationship between the lesion location of acute ischemic stroke and early depressive symptoms in Japanese patients. Ann Gen Psychiatry (2016) 15:12. 10.1186/s12991-016-0099-x 27042194 PMC4818403

[57] Robinson RG Price TR. Post-stroke depressive disorders: a follow-up study of 103 patients. Stroke (1982) 13:635–41. 10.1161/01.STR.13.5.635 7123596

[58] Parikh RM Lipsey JR Robinson RG Price TR. Two-year longitudinal study of post-stroke mood disorders: dynamic changes in correlates of depression at one and two years. Stroke (1987) 18:579–84. 10.1161/01.STR.18.5.837 3590249

[59] Shi YZ Xiang YT Wu SL Zhang N Zhou J Bai Y The relationship between frontal lobe lesions, course of post-stroke depression, and 1-year prognosis in patients with first-ever ischemic stroke. PLoS One (2014) 9:e100456. 10.1371/journal.pone.0100456 25003990 PMC4086722

[60] Robinson RG Starr LB Kubos KL Price TR. A two-year longitudinal study of post-stroke mood disorders: findings during the initial evaluation. Stroke (1983) 14:736–41. 10.1161/01.STR.14.5.736 6658957

[61] Starkstein SE Robinson RG Price TR. Comparison of cortical and subcortical lesions in the production of poststroke mood disorders. Brain (1987) 110:1045–59. 10.1093/brain/110.4.1045 3651794

[62] Vataja R Leppävuori A Pohjasvaara T Mäntylä R Aronen HJ Salonen O Poststroke depression and lesion location revisited. J Neuropsychiatry Clin Neurosci (2004) 16:156–62. 10.1176/appi.neuropsych.16.2.156 15260366

[63] Singh A Herrmann N Black SE. The importance of lesion location in poststroke depression: a critical review. Can J Psychiatry (1998) 43:921–7. 10.1177/070674379804300907 9825164

[64] Gozzi SA Wood AG Chen J Vaddadi K Phan TG. Imaging predictors of poststroke depression: methodological factors in voxel-based analysis. BMJ Open (2014) 4:e004948. 10.1136/bmjopen-2014-004948 25001395 PMC4091263

[65] Rorden C Brett M. Stereotaxic display of brain lesions. Behav Neurol (2000) 12:191–200. 10.1155/2000/421719 11568431

[66] Medina J Kimberg DY Chatterjee A Coslett HB Inappropriate usage of the Brunner–Munzel test in recent voxel-based lesion-symptom mapping studies. Neuropsychologia (2010) 48:341–3. 10.1016/j.neuropsychologia.2009.09.016 19766664 PMC2795086

[67] Mah Y-H Husain M Rees G Nachev P. Human brain lesion-deficit inference remapped. Brain (2014) 137(Pt 9):2522–31. 10.1093/brain/awu164 24974384 PMC4132645

[68] Hama S Murakami T Yamashita H Onoda K Yamawaki S Kurisu K Neuroanatomic pathways associated with monoaminergic dysregulation after stroke. Int J Geriatr Psychiatry (2016) 32(6):633–42. 10.1002/gps.4503 27251297

[69] Basser PJ Pierpaoli C Microstructural and physiological features of tissues elucidated by quantitative-diffusion-tensor MRI. J Magn Reson (1996) 111:209–19. 10.1016/j.jmr.2011.09.022 8661285

[70] Chen G Hu X Li L Huang X Lui S Kuang W Disorganization of white matter architecture in major depressive disorder: a meta-analysis of diffusion tensor imaging with tract-based spatial statistics. Sci Rep (2016) 6:1–11. 10.1038/srep21825 26906716 PMC4764827

[71] Bhogal SK Teasell R Foley N Speechley M. Lesion location and poststroke depression: systematic review of the methodological limitations in the literature. Stroke (2004) 35:794–802. 10.1161/01.STR.0000117237.98749.26 14963278

